# Can teaching hospitals use serial formative OSCEs to improve student performance?

**DOI:** 10.1186/s13104-016-2266-1

**Published:** 2016-10-14

**Authors:** Heng-Hui Lien, Sang-Feng Hsu, Shu-Chen Chen, Jiann-Horng Yeh

**Affiliations:** 1 School of Medicine, Fu Jen Catholic University, New Taipei City, Taiwan; 2 Clinical Skills Center, Cathay General Hospital, 280 Sec.4, Jen-Ai Road, Taipei, Taiwan

**Keywords:** Clinical skill, OSCE

## Abstract

**Background:**

We report on interns’ clinical competence and experiences of an objective structured clinical examination (OSCE) training program over 3 years. We aimed to determine whether repeated formative OSCEs allow teaching hospitals to improve the effectiveness of clinical training and help interns to achieve high scores in the national summative OSCE.

**Methods:**

This study included 207 participants, among whom 82 were interns who had completed four mock OSCEs and a national OSCE at the clinical center of Cathay General Hospital (CGH). The other 125 participants were final-year medical students from Fu-Jen University who had completed the national OSCE between 2013 and 2015 at one of four teaching hospitals (including CGH). CGH interns were categorized into three groups according to the medical school attended and Fu-Jen University students were grouped according to their training hospitals. CGH held four mock OSCEs (30 stations), whereas each of the four training hospitals for Fu-Jen students each held one or two OSCEs (6–12 stations) annually. Differences in the mean OSCE scores among groups were analyzed. The medical school attended, pre-internship OSCE experience and the frequency of mock OSCEs held by training hospitals were independent factors in this study.

**Results:**

The cumulative mean scores for five OSCEs among three groups of students trained at CGH tended to increase from the first OSCE (OSCE1) to the fifth (OSCE5). The mean score of the students who attended Fu-Jen Medical School was higher than that of students who graduated from foreign medical schools in all five OSCEs; however, the differences were significant only for OSCE2 (P = 0.022) and OSCE3 (P = 0.027). The mean national OSCE scores of FJU students showed no statistically significant differences among the four training hospitals for 2013; however, students training at CGH had significantly higher mean scores in the 2014 (P = 0.001) and 2015 (P = 0.005) OSCEs compared with students training at the other three hospitals.

**Conclusions:**

Serial administration of formative OSCEs by teaching hospitals enhances the performance of students on the sequential summative OSCE. Such programs provide multiple opportunities for students to practice their clinical skills, and for faculty to develop their teaching, assessment and consensus building skills.

## Background

Administration of formative and summative objective structured clinical examinations (OSCEs) in teaching programs has been shown to improve final-year medical school student’s examination performance [1]. Although the correlation between clinical competence and high-stakes OSCEs is widely accepted [2, 3], it remains unclear whether serial administration of formative OSCEs in clinical teaching programs leads to consistent improvement of clinical performance. Therefore, in this study, we investigated whether serially administered formative OSCEs help medical students achieve high scores in the national OSCE.

OSCEs are well-established as effective assessment tools for clinical competence. As such, they have become an important part of the medical licensing process in many countries [4, 5]. In Taiwan, the Taiwan Association of Medical Education, jointly commissioned by the Examination Yuan, Ministry of Education and Ministry of Health and Welfare, established an OSCE office to initiate a national medical licensure OSCE program. Passing the national OSCE has been a requirement for advancement to the second-stage national medical licensing exam in Taiwan since 2013 [6]. The Taiwanese national OSCE was held in over 20 certified examination units set in teaching hospitals or medical schools.

OSCE-related policies also influence various aspects of the medical education systems that apply OSCEs as a formative educational mode [7]. All students are repeatedly instructed about the goals of OSCEs. During formative OSCEs, students are given the opportunity to interact with standardized patients (SPs), and raters (their teachers) provide immediate feedback at each station. Serially administered OSCEs increase the reliability of borderline students through increased practice time [8]. Such programs can narrow the clinical skills gap among students with different training backgrounds. Such activities also enhance the evaluation and teaching abilities of teachers, helping to create a positive atmosphere in education systems. Hence, investigating the response of students completing serial mock OSCEs at training hospital should help determine the examinations’ influence students’ performance of clinical skills. Our study analyzed the annual performance of 82 final-year medical students of Cathay General Hospital (CGH) who completed five OSCEs, the fifth of which was the national OSCE held in CGH, and 125 final year medical students from Fu-Jen University (FJU) who completed national OSCEs, which were held in CGH and three other teaching hospitals (herein referred to as hospitals F, S, K) during 2013–2015.

## Methods

This retrospective study was approved by the Institutional Review Board (IRB) of Cathay General Hospital (CGHIRB No: CGH-P104084). The IRB granted a waiver of informed consent from the participants in this study; their records were anonymized and de-identified prior to analysis. Eighty-two students who received clinical training at CGH between 2013 and 2015 were categorized according to the medical school attended into three groups labelled A, B and C. Group A comprised students from FJU (n = 31); group B contained students from medical school B of Taiwan (n = 30); and group C comprised graduates from foreign medical schools (n = 21). The students in all the groups had completed a set of five OSCEs within 1 academic year between 2013 and 2015; the first four were formative OSCEs in which station raters provided students with direct feedback, and the final examination was the national OSCE. Individual student scores on each OSCE were recorded and the differences in the mean scores among groups A, B and C were analyzed. Regarding previous (i.e., pre-internship) experience of OSCEs, participants in group A completed OSCEs six times on average, whereas those in Group B had completed them once and those in Group C had no experience of OSCEs. The first to fourth mock OSCEs (OSCE1–OSCE4) were held during the months of October, December, February, and April, respectively, and the final national OSCE (OSCE5) was held in the last week of April. Three OSCEs (OSCE1, OSCE2, and OSCE4) contained six stations (four SP stations and two procedure operation stations) and two OSCEs (OSCE3 and OSCE5) comprised 12 stations (eight SP stations and four procedure operation stations). The allocated fields for each OSCE station covered five departments: internal medicine, surgery, gynecology, pediatrics, and emergency. These fields correspond to five categories of clinical skills: history taking, physical examination, explanation and management, communication and counseling, and procedure skills. Students were required to achieve not only the passing score at a given minimum number of stations (7 stations) but also the overall passing score. The passing score for each station was calculated using borderline-group and the borderline regression methods. Each station in OSCE1–OSCE4 involved 8 min of observation and 2 min of immediate verbal feedback from station raters. Students received formal individualized performance analysis reports, and participated in a 90-min discussion meeting 1 week after each mock OSCE to review the correct procedures for each station with the aim of improving performance at the next examination. The last national OSCE (OSCE5) was a summative examination; students were given 8 min at each station. All raters were clinical physicians with actual scoring experience as certified by the OSCE office. All SPs were certified as having performance experience. Discrimination, difficulty and passing score of each station were calculated and compared according to guidelines set out by the OSCE office.

The 125 final-year FJU medical students received clinical training at four teaching hospitals: F hospital (FH), S hospital (SH), K hospital (KH) and CGH. Each student in this group had participated in the national OSCE (the fifth OSCE of CGH) between 2013 and 2015. The mean scores of these students in each teaching hospital were compared. CGH held four mock OSCEs (30 stations), whereas the remaining teaching hospitals held one or two mock OSCEs (6–12 stations) annually.

### Statistical methods

An analysis of variance (ANOVA) was performed to compare the differences among the study groups. A post hoc analysis was subsequently conducted for the groups showing significant differences; given that the sizes of the groups differed, the Scheffe method was used to prevent type-one error.

## Results

### Student performance

**Fig. 1:**
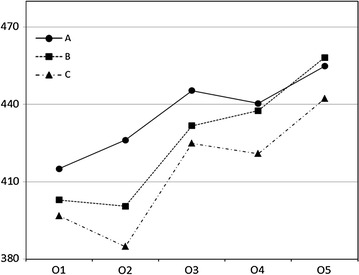
OSCE1–OSCE5 mean scores for group **a**, **b** and **c** students (N = 82) trained at CGH between 2013 and 2015; OSCE3 and OSCE5 mean scores were adjusted

For the 82 students trained at CGH from 2013 to 2015, the cumulative mean scores for OSCE1 through OSCE5 showed positive curve (the scores for OSCE3 and OSCE5 were adjusted because they each contained 12 stations, whereas the others only had six stations) (Fig. 1). Among the three groups, group A had a higher mean score in the five CGH OSCEs compared with group C. ANOVA showed significant differences for OSCE2 and OSCE3; however, no significant differences were found for OSCE1, OSCE4, and OSCE5 (Table 1). Post-hoc analysis for groups A, B and C revealed significant differences between groups A and C in OSCE2 and OSCE3 scores (OSCE2, P = 0.022; OSCE3, P = 0.027) (Table 2).

**Table 1:** ANOVA test results of difference in mean scores from OSCE1–OSCE5 for CGH student groups A, B and C, 2013–2015

	Group A; n = 31		Group B; n = 30		Group C; n = 21		ANOVA test
	Mean	SD	Mean	SD	Mean	SD	P value
OSCE1	415	35.5	403.0	41.2	396.9	38.3	0.261
OSCE2	426.2	43.1	400.6	41.0	394.3	43.3	0.022*
OSCE3	890.7	55.0	863.4	60.1	849.9	45.8	0.027*
OSCE4	440.4	40.3	437.6	45.2	420.9	39.5	0.236
OSCE5	909.7	46.5	916.3	66.5	884.5	41.2	0.104

* P < 0.05

**Table 2:** Post-hoc test results of differences in mean scores from OSCE1–OSCE5 for CGH student groups A, B and C, 2013–2015

Post hoc		Diff	Lower	Upper	P value
OSCE2					
Group A–group B		25.601	−1.780	52.981	0.072
Group C–group B		−6.275	−36.224	23.673	0.871
Group C–group A		−31.876	−61.596	−2.156	0.033*
OSCE3					
Group A–group B		27.292	−6.534	61.117	0.138
Group C–group B		−13.519	−50.792	23.755	0.663
Group C–group A		−40.810	−78.084	−3.536	0.028*

* P < 0.05

### Student performance over time

The mean scores of the 125 FJU students in the national OSCEs of 2013–2015 were analyzed using ANOVA. The 2013 results revealed no significant differences; FH-trained students registered the highest mean score, followed by SH, KH, and CGH. The differences for 2014 and 2015 were all significant (Table 3). For the 2014 test, CGH demonstrated the highest mean score, followed by FH, SH, and KH. Post-hoc analysis revealed statistically significant differences between CGH and FH (P = 0.018), FH and KH (P = 0.003), CGH and SH (P < 0.001), and CGH and KH (P < 0.001) (Table 4). Furthermore, for the 2015 test, CGH-trained students registered the highest mean score, followed by KH, FH and SH. Post-hoc analysis showed significant differences between the mean scores of CGH and SH (P = 0.002) (Table 4).

**Table 3:** ANOVA test results of difference in mean scores from national OSCE for final-year FJU medical students at FH, SH, KH and CGH, 2013–2015

2013	FH		SH		CGH		KH		ANOVA
	n = 12		n = 10		n = 9		n = 5		P
	Mean	SD	Mean	SD	Mean	SD	Mean	SD	
	926.5	45.9	905.0	38.5	887.9	44.4	888.7	42	0.185

2014	FH		SH		CGH		KH		ANOVA
	n = 16		n = 11		n = 10		n = 8		P
	Mean	SD	Mean	SD	Mean	SD	Mean	SD	
	871.7	44.0	828.8	46.2	932.4	49.8	792.0	58.7	0.001*

2015	FH		SH		CGH		KH		ANOVA
	n = 12		n = 11		n = 13		n = 8		P
	Mean	SD	Mean	SD	Mean	SD	Mean	SD	
	874.8	61.0	819.3	57.5	908.2	38.3	875.4	71.5	0.005*

* P < 0.05

**Table 4:** Post-hoc test results of difference in mean scores from national OSCE for final-year FJU medical students at FH, SH, KH and CGH, 2013–2015

Post hoc	Diff	Lower	Upper	P value
2013				
S-F	−21.532	−71.508	28.445	0.651
CGH-F	−38.646	−90.114	12.823	0.197
K-F	−37.869	−99.997	24.260	0.365
CGH-S	−17.114	−70.743	36.515	0.823
K-S	−16.337	−80.267	47.593	0.899
K-CGH	0.777	−64.326	65.880	1.000
2014				
S-F	−42.914	−93.865	8.038	0.126
CGH-F	60.714	8.275	113.154	0.018*
K-F	−79.718	−136.047	−23.389	0.003*
CGH-S	103.628	46.789	160.467	<0.001*
K-S	−36.804	−97.250	23.642	0.373
K-CGH	−140.433	−202.138	−78.727	<0.001*
2015				
S-F	−55.431	−118.563	7.702	0.103
CGH-F	33.450	−27.096	93.995	0.458
K-F	0.672	−68.361	69.705	1.000
CGH-S	88.880	26.920	150.840	0.002*
K-S	56.103	−14.174	126.379	0.158
K-CGH	−32.778	−100.740	35.185	0.573

* P < 0.05

## Discussion

The differences in the mean scores for the five OSCEs among group A, B and C CGH students were significant for OSCE2 and OSCE3 but not for OSCE1, OSCE4 and OSCE5. There was a gradual but observable increase on the mean learning curve, consistent with previously reported results [9]. Medical school attended and pre-internship OSCE experience did not affect student performance in OSCE1; however, these factors affected performance in OSCE2 and OSCE3. Unfamiliarity with the format of the OSCE and/or its related clinical competencies was a major factor affecting performance in OSCE1. Medical school attended (group C) and pre-internship OSCE experience (group A) affected students’ performance in OSCE2 and OSCE3 when the influence of unfamiliarity was attenuated. There was no significant difference in performance among the three groups in OSCE4 and OSCE5. Taken together, these findings demonstrate that, after three mock OSCEs, the influence of the abovementioned factors (i.e., unfamiliarity with the OSCE, medical school attended, and pre-internship OSCE experience) seems attenuated, enabling students to show consistent performance levels.

Unfamiliarity with OSCE format not only interferes with the performance of students but also with that of staff at the training hospitals wherein OSCEs are held. To reduce the risk of unfamiliarity, high-stakes national OSCEs were administered in Taiwan in 2011 and 2012, before the examination became one of the formal criteria of medical licensure in 2013. The differences in the mean scores from the 2013 national OSCE of the FJU students training at the four hospitals were not significant. All raters, SPs and station developers had to become familiar with the test through repeated practice. The differences in the mean scores on the national OSCE among the four teaching hospitals were significant in 2014 and 2015. In these tests, hospitals incorporating more mock OSCEs, such as CGH, registered higher mean scores. Students of CGH gained more experience from the four mock OSCEs (30 stations) than did students at the other hospitals. The stations in these mock examinations featured SPs in well-designed clinical scenarios, and raters, who provided immediate feedback. Such a training style is highly beneficial for faculty development and student training [10]. The mock OSCE training program builds various domains of medical education in an integrated, coherent, and longitudinal fashion, and provides students with frequent and constructive feedback [11]. Because OSCEs are becoming more widely incorporated, future work should examine the potential of applying OSCEs at different stages of medical training for predicting and improving clinical performance [12]. In line with Ericsson’s concept of deliberate practice, the repeated practice the mock OSCEs afforded and the corrective feedback received from tutors likely contributed to students’ better performance [13, 14].

## Conclusions

A limitation of this study was the small sample size. However, OSCE training programs are easier to manage when conducted on small groups comprising approximately 30 students. The 2015 CGH OSCE training program, for example, required two workshops (one for raters and one for SPs), 122 raters, 75 SPs and 54 OSCE stations to train only 33 students. All methods for the training of raters and SPs, development of OSCE stations, and administration of OSCEs were qualified and followed the standards set by the OSCE office. Fine tuning of this training program is essential for application in teaching hospitals of different sizes. The administration of mock OSCEs by teaching hospitals enhances the performance students on the summative OSCE, as well as the teaching and assessment abilities of teachers and program directors.

## Abbreviations

OSCE: objective structured clinical examinations
CGH: Cathay General Hospital
FJH: Fu-Jen University
FH: F hospital
SH: S hospital
KH: K hospital
